# Effects of human-driven water stress on river ecosystems: a meta-analysis

**DOI:** 10.1038/s41598-018-29807-7

**Published:** 2018-07-30

**Authors:** Sergi Sabater, Francesco Bregoli, Vicenç Acuña, Damià Barceló, Arturo Elosegi, Antoni Ginebreda, Rafael Marcé, Isabel Muñoz, Laia Sabater-Liesa, Verónica Ferreira

**Affiliations:** 1 Catalan Institute for Water Research (ICRA), Carrer Emili Grahit 101, 17003 Girona, Spain; 2 Institute of Aquatic Ecology, University of Girona, Campus Montilivi, 17071 Girona, Spain; 3 Water Science and Engineering Department, IHE Delft Institute for Water Education, Westvest 7, 2611 AX Delft, The Netherlands; 4 Department of Environmental Chemistry, Institute of Environmental Assessment and Water Research (IDAEA-CSIC), Carrer Jordi Girona 18-26, 08034 Barcelona, Spain; 5 Laboratory of Stream Ecology, Department of Plant Biology and Ecology, University of the Basque Country, 48080 Bilbao, Spain; 6 Department of Evolutionary Biology, Ecology and Environmental Sciences, Universitat de Barcelona, Avgda. Diagonal 643, 08028 Barcelona, Spain; 7 MARE – Marine and Environmental Sciences Centre, Department of Life Sciences, University of Coimbra, 3004-517 Coimbra, Portugal

## Abstract

Human appropriation of water resources may induce water stress in freshwater ecosystems when ecosystem needs are not met. Intensive abstraction and regulation cause river ecosystems to shift towards non-natural flow regimes, which might have implications for their water quality, biological structure and functioning. We performed a meta-analysis of published studies to assess the potential effects of water stress on nutrients, microcontaminants, biological communities (bacteria, algae, invertebrates and fish), and ecosystem functions (organic matter breakdown, gross primary production and respiration). Despite the different nature of the flow regime changes, our meta-analysis showed significant effects of human-driven water stress, such as significant increases in algal biomass and metabolism and reduced invertebrate richness, abundance and density and organic matter decomposition. Water stress also significantly decreased phosphate concentration and increased the concentration of pharmaceutical compounds. The magnitude of significant effects was dependent on climate, rainfall regime, period of the year, river size and type of water stress. Among the different causes of water stress, flow regulation by dams produced the strongest effects, followed by water abstraction and channelization.

## Introduction

The use of water resources is one of the strongest manifestations of nature-human cross-linkages^1^ and is likely to increase due to the rising human population, climate change and land use changes^2^. The intensive use of water resources may lead to a structural deficit or water scarcity^3^, affecting the economic development of nearly 1.4 billion people^1,4^ and even compromising human health^5^. In addition to social implications, human appropriation of water resources may induce water stress in freshwater ecosystems^6^, i.e., changes in quantity (over-exploitation and altered flow regimes) and quality (excess nutrient, pollution and less biodiversity) beyond their natural variability.

Watercourses are intensively managed in many areas of the world, especially in regions where water is scarce^7–9^. Weirs, dams, channelisation, groundwater exploitation and direct water abstraction are common practices, primarily aimed at supplying water for agricultural, urban and industrial purposes. In these situations, altered flow regimes subsequently affect water quality and biodiversity. This human-driven water stress (HDWS) differs from naturally-occurring water stress of intermittent or temporary rivers, which characteristically show a decreased or interrupted flow for given period(s) of the year^10^. Flow reduction or cessation is predictable in intermittent or temporary rivers^11^ and is usually associated with climate variability. The biological communities of temporary rivers are usually adapted to these changes, displaying higher resistance and resilience^12^. However, HDWS causes unprecedented flow regime alterations, occurring at any time of the hydrological cycle based on human management^13–15^. The resulting anomalous flow regimes may therefore impact on non-adapted biological communities. Furthermore, natural flow decrease or cessation in temporary rivers follows characteristic spatial patterns depending on the intensity of the dry period^16^, whereas changes in the flow regimes produced by HDWS are spatially related to water infrastructures and cause a contrasting situation of decreased water flow and/or an altered hydrograph downstream^17^.

In arid or semi-arid river ecosystems, HDWS may cause so-called “artificial droughts”^16^ or human-induced water flow intermittency. Even under less severe situations, altered flow regimes cause a certain degree of water stress^18–20^ that reduces natural dynamism, but not to the extreme of drying out. In general, water-stressed systems are characterised by longer low-flow periods and less frequent and smaller peak flows^21–23^, favouring hydrological stability instead of the natural dynamism typical of river ecosystems^24^.

HDWS may have significant effects on freshwater ecosystems. The concentration of nutrients and pollutants may follow particular patterns^25,26^, with reduced peak flows affecting in-stream habitats and sediment transport^17,27,28^. This could in turn affect the composition, abundance and diversity of biological communities, although the common directions of these effects are still unclear^29–31^. At least in some cases, altered hydrographs may promote the accumulation of primary producers on the streambed, increasing ecosystem metabolism^32,33^. However, the response patterns may diverge between river ecosystems. Particular environmental conditions of the river or even the source of water stress might produce different outcomes. For example, effects are more severe in naturally arid or semi-arid systems (e.g., Mediterranean) than in humid ones (e.g., Atlantic or Continental) where water flow changes are less substantial^14,15^. As such, studies do not support univocal patterns and reflect a large diversity of responses. This variability might be due to the described environmental conditions and also to the few cases described in most papers that make the intensity and prevalence of effects difficult to generalise.

In this study, we reviewed the current literature and analysed the components and functions of river ecosystems affected by HDWS. We performed a meta-analysis to identify central trends across multiple case studies and assess the significance, magnitude and direction of effects of water stress on water quality (i.e., concentration of nutrients and microcontaminants), biological communities (the abundance, biomass and diversity of bacteria, algae, invertebrates and fish) and river ecosystem functions (primary production, respiration and organic matter decomposition). We also aimed to identify the factors that might influence the magnitude and direction of the effects, as well as any gaps in research. Outlining these patterns may help to forecast and mitigate the effects produced by global environmental changes on river ecosystems.

## Methodology

### Literature search and study selection

We completed a bibliographic search on May 2017, using ISI and Google Scholar, to retrieve referenced and non-referenced publications in English without time restrictions. The publications had to report the effect of HDWS on river water characteristics, biota or ecosystem functions. We used an integrative list of terms describing water stress: *water scarcity*, *water stress*, *flow intermittency*, *flow regulation*, *dam*, *water abstraction*, *low flow*, and *basal flow*, together with their derivatives combined with *river* OR *stream*. These terms were used in combination with other terms (and their derivatives) such as (1) biogeochemical terms or contaminants: *nutrient* OR *nitrogen* OR *phosphorus* OR *total phosphorus* OR *dissolved inorganic nitrogen* (*DIN)*; *micropollutants* OR *microcontaminants* OR *organic pollutant* OR *emerging pollutant* OR *pharmaceutical products* OR *personal care products* OR *pesticides* OR *endocrine disruptor* OR *perfluorinated compounds* OR *illicit drugs*; (2) the main biological groups in river systems: *bacteria*; *algae* OR *biofilm* OR *periphyton*; *invertebrate*; *fish*; (3) the most relevant riverine ecosystem functions: *organic matter decomposition* OR *litter* OR *leaf breakdown* OR *decay*; *metabolism* OR *gross primary production* OR *respiration* OR *nutrient uptake*. We also surveyed the reference lists of relevant publications for additional references.

This search yielded over 1,000 papers, which were individually assessed and selected if the following criteria were met: (a) quantitative data were available from which an average value, an estimate of data variability and sample size could be obtained from both a control (non-HDWS) and an impacted site; (b) information on the type of human impact (dam, water diversion, channelisation or groundwater exploitation) was available. This resulted in 44 relevant studies (Table S1), the majority comparing an upstream Control site with a downstream Impacted site (e.g., Menéndez *et al*. 2012) and 262 Control-Impact comparisons (Table S2). In the case of before-after-control-impact (BACI) studies, the control and impact data were obtained from the *after* period^13^. A few studies had temporal data from a given site that had experienced changes in its level of water stress with time (Before (~Control) *vs* After (~Impact)^34^).

### Data extraction

Data on sample size, means and measures of variability were extracted directly from tables, obtained directly from the authors or (in a few cases) extracted from figures using the WebPlotDigitizer version 3.8 software. The mean values of water characteristics, biota and/or ecosystem functions in Control and Impacted conditions were collected initially in all available units; however, the final decision on the data to be used was based on the most common variables or units used in order to obtain significant numbers (n > 2) for statistical analyses. Variability measures included standard deviation (SD), standard error (SE) or the 95% confidence limit (CL), with the SE and 95% CL being converted into SD for the analysis (Table S2).

Additionally, we collected information on moderator variables (i.e., variables that could explain differences in the effects of HDWS across studies): period of the year, climate (e.g., Atlantic or Tropical), rainfall regime (e.g., humid or dry), river size, nutrient status, the type of water stress (e.g., damming), and the presence of waste water treatment plants (WWTP). This information was used as categorical values (Tables 1 and S2). We collected hydrological data (average water flow and the temporal variability in water flow in the Control and Impacted sites), when available, to define the extent of water stress in the Impacted site compared to the Control site. Since these data were not available in the majority of studies, it could not be directly used as a moderator in the analysis.

**Table 1:** Identification, levels and definition of moderators used in the analyses and percentage of case studies per moderator level.

Moderator	Levels	Definition	% distribution in the matrix
Paper	Several	Identifies the primary paper.	
		*Full references are given in Table* S1	
*Variable type*	Chemical	Contaminants and nutrients	26
	Bacteria	Biofilm bacteria	10.7
	Algae	Benthic river algae	19.1
	Invertebrates	River macroinvertebrates	18.3
	Fish	River fish	5
	Function	Metabolism and decomposition of organic matter	21.0
*Variable type 2*	Nutrients	Phosphorus and nitrogen forms	21.4
	Pharmaceuticals	concentration and number	2.3
	PersonalCareProducts	concentration and number	0.8
	Pesticides	concentration and number	0.8
	Perfluorates	concentration and number	0.8
	Metabolism	Gross primary production and Respiration	5.3
	Breakdown	decay rate of organic matter	15.6
Period of the year	winter		14.9
	spring		17.9
	summer		34.4
	autumn		25.2
	annual		15.3
Climate	temperate	Temperate (excluding Mediterranean)	34.0
	mediterranean	Mediterranean-like climates	46.9
	tropical	Tropical and Equatorial climates	3.1
	continental	Atlantic-like climates	16.0
Rainfall regime	arid	Very poor rainfall	2.3
	semiarid	Moderate rainfall	42.4
	humid	High rainfall	55.3
River size	small	Headwaters	30.9
	medium	Middle courses	24.8
	large	Low watercourses	37.4
Water stress type	dam	River regulated by a dam	57.6
	diversion	Existing water diversion	34.7
	groundwater expl.	Existing groundwater abstraction	6.1
	channelization	Existing channelization	1.9
	undetermined	Unspecific cause of water stress	1.1
Nutrient status	nutrient-poor	Oligotrophic systems	59.5
	nutrient-rich	Eutrophic systems	30.2
WWTP	presence	presence of waste water treatment plant	5.0
	absence	absence of waste water treatment plant	95.0

### Effect size

The effect size of HDWS was calculated as the response ratio (R), i.e., the ratio of the variable of interest at the Impacted condition to the variable of interest at the Control condition (R = Impacted:Control^35^). R = 1 indicated no effect of water stress, R < 1 indicated an inhibition or decrease and R > 1 indicated a stimulation or increase of the variable of interest due to water stress. Values were ln-transformed (lnR) for the analyses (Table S2). The variance associated with the effect size (V_lnR_), which is needed to weigh each effect size by its precision, was estimated from the SD and sample size of each mean value^36^.

Many studies contributed multiple effect sizes to the matrix when they reported the response of multiple variables to water stress (e.g., water chemistry and biological variables^13,14^), the effects of water stress under several conditions (e.g., different seasons or nutrient status;^28^) or considered multiple Control – Impacted comparisons^32,37^. Although several cases originating from the same study may not be independent, not considering them would have restricted our analysis. We therefore included these in the analysis, but assessed their effect on the results by sensitivity analysis (see below).

### Statistical analysis

Analyses were performed in OpenMEE^38^. The grand mean effect size, i.e., the overall response of the variable of interest to water stress, was determined using a random-effects model of meta-analysis. Between-study variance was estimated using the restricted maximum likelihood (REML) method. The mean effect size for each variable of interest (water chemistry, biological variables and ecosystem functions) was also assessed (random-effects model and REML) and compared by sub-group analysis^36,38^. The effects of the moderator variables on the magnitude and direction of the response of the variables of interest to water stress were also assessed by sub-group analysis for subsets of the matrix according to our hypotheses (see Introduction) and available sample size; only levels with n > 2 were compared. Analyses were performed in lnR and results were back transformed to R to facilitate interpretation. Effects were significant if the 95% CL did not include 1 and the effects were significantly different between levels within a given moderator if their 95% CL did not overlap^36^. The percentage of total variability caused by between-study variation rather than sampling error (I^2^) was also calculated^36^.

### Publication bias

The robustness of the entire matrix or subsets of the matrix to publication bias (e.g., the selective publication of studies with significant effects over those not finding significant effects) was tested by the Rosenberg fail-safe number, which gives the number of missing Control – Impacted comparisons (or studies in the case of sensitivity analyses) with non-significant results that would be needed to nullify the combined effect size. If the fail-safe number (Nfs) is high (>5 × n + 10, where n = number of Control – Impacted comparisons), the results can be considered robust despite the possibility of publication bias^36^.

### Sensitivity analysis

The effect of considering multiple Control – Impacted comparisons from each study on the results was assessed by sensitivity analysis. The analyses were repeated to the greatest extent possible, considering a mean effect size per study-variable combination, which was calculated as the weighed mean effect size of all the Control – Impacted comparisons considered within that study-variable (i.e., study-variable was considered as the grouping variable in a subgroup analysis).

### Availability of materials and data

Authors make all the materials and data used in the paper available to readers, without restrictions. This manuscript contains supporting materials in the form of supplementary data.

## Results

### Database

The obtained biological descriptors (53.1% of the effect sizes) included bacteria (density and enzymatic activities; 10.7% of the data), algae (biomass; 19.1% of the data), invertebrates (abundance, density, richness and diversity; 18.3% of the data) and fish (density; 5% of the data) (Table 1). Chemical variables were noted in 26% of the data, with nutrients (total and reactive phosphorus, ammonia, nitrate and DIN) contributing 21.4% of the data. Among the microcontaminants (4.7% of the data; pharmaceutical products, pesticides, personal care products and industrial compounds), only pharmaceutical products were represented by >2 data (Table 1). Among the ecosystem functions (21% of the data), river metabolism accounted for 5.4% of the data (2.7% for gross primary production and 2.7% for ecosystem respiration) and organic matter breakdown 15.3% (Table 1). Most of the papers selected for the meta-analysis investigated systems in Mediterranean-like climates (46.9%), although 34% included temperate non-Mediterranean climates (Table 1). Most of the available data were from humid areas (55.3%), while sites with low rainfall accounted for only 2.3% of the total data. Most of the collected data were obtained in summer (34.4%), although a large amount consisted of annual records. A third of the data came from studies performed in low-order streams, the rest from between middle-sized and large rivers. Most of the data were from nutrient-poor systems (59.5%) and the majority was not affected by WWTP effluents (95%). The main cause of water stress was the presence of dams (57.6%), followed by water diversion (34.7%); a small number of records were obtained from studies investigating the effects of groundwater extraction (6.1%) or channelization (1.9%).

The effect of water regulation could be calculated in the studies reporting discharge values upstream and downstream of a dam (n = 47). The reduction in discharge was 41.7 ± 47.1% (average ± SD, range 6–98%) with respect to the Control site. In some studies from humid areas (n = 5), water flow was higher downstream of the dam. Regulation reduced flow variability to between 32.8 and 96% with respect to the Control site (n = 16).

### Overall effects of water stress

**Figure 1:**
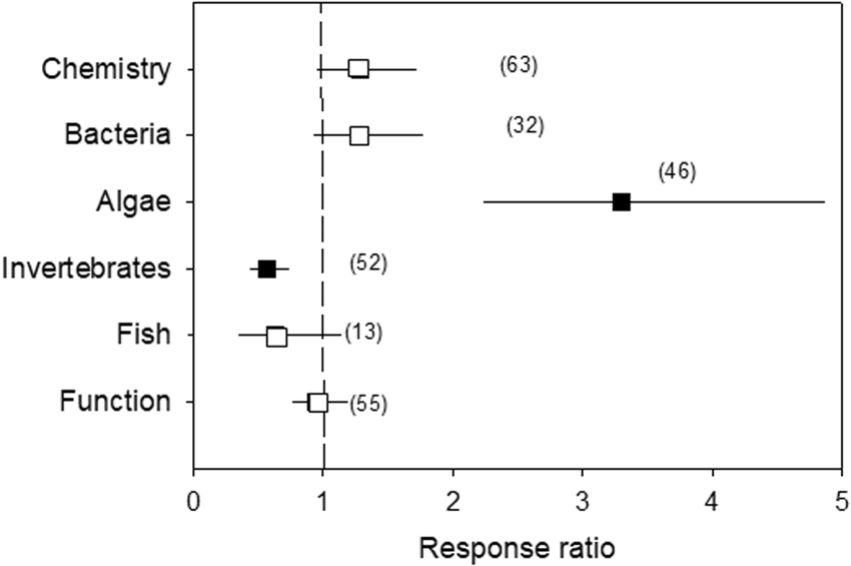
Effects of water stress on water chemistry, biota (bacteria, algae, invertebrates and fish) and ecosystem function, given by the response ratio (R = Impacted/Control; ±95% Confidence Limit, CL). The dashed line (mean effect size = 1) indicates no effect. Mean effect size >1 indicates an increase, while mean effect size <1 indicates a decrease due to water stress. The effect of water stress is significant when the 95% CL does not overlap 1 (black symbols). Variables do not significantly differ when their 95% CL overlap. Values in the parentheses indicate sample size.

Water stress significantly enhanced algal biomass (R = 3.30; 95% CL: 2.24–4.86), decreased invertebrate variables (R = 0.56; 95% CL: 0.43–0.73), and had no significant effect on water chemistry (R = 1.28; 95% CL: 0.95–1.72), bacteria (R = 1.28; 95% CL: 0.93–1.77), fish (R = 0.63; 95% CL: 0.35–1.14) and ecosystem functions (R = 0.95; 95% CL: 0.76–1.19) (Fig. 1). Subsets of the variables assessed (water chemistry, biological variables and ecosystem function), except fish (low sample size) and bacteria (not free from publication bias; Nfs > threshold), were analysed further to identify the moderators of the effect of water stress (see below).

### Water chemistry

**Figure 2:**
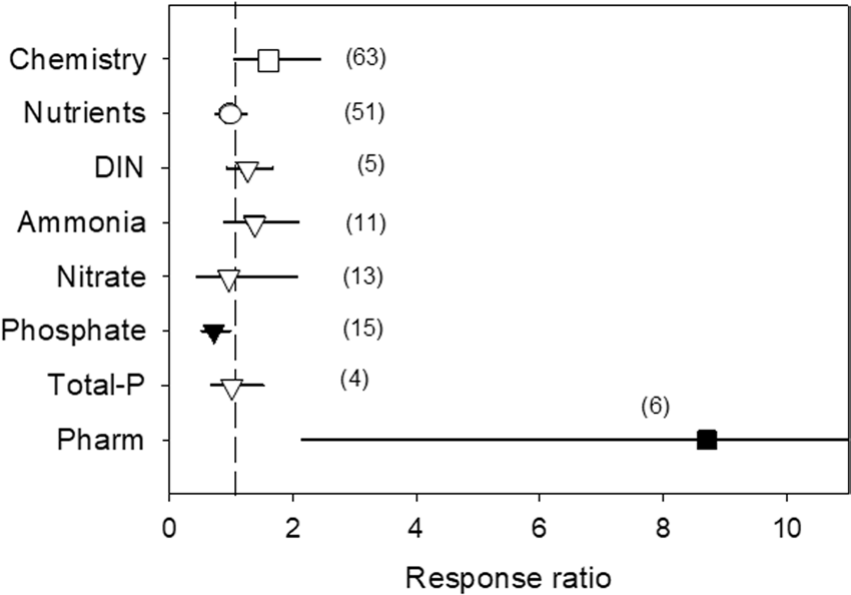
Effects of water stress on chemical descriptors, given by the response ratio (R = Impacted/Control; ±95% Confidence Limit, CL). The dashed line (mean effect size = 1) indicates no effect. Mean effect size >1 indicates an increase, while mean effect size <1 indicates a decrease due to water stress. The effect of water stress is significant when the 95% CL does not overlap 1 (black symbols). Levels within a given moderator (same symbol) do not significantly differ when the 95% CL overlap. Values in the parentheses indicate sample size. DIN, dissolved inorganic nitrogen; Pharm, pharmaceutical compounds.

For water chemistry, the two groups of variables (nutrients and pharmaceutical products) showed different responses. Pharmaceutical product concentrations were significantly affected by HDWS (8.71-fold increase), but variation was large (95% CL: 2.15–35.30) probably due to the small sample size (Fig. 2). For the nutrients, only PO_4_ concentration was significantly affected by HDWS, showing a reduction of 27% (R = 0.73; 95% CL: 0.53–0.98). NH_4_, NO_3_, DIN and total-P concentrations were not significantly affected by HDWS, but sample sizes were small and/or variation was large (Fig. 2).

### Algae

**Figure 3:**
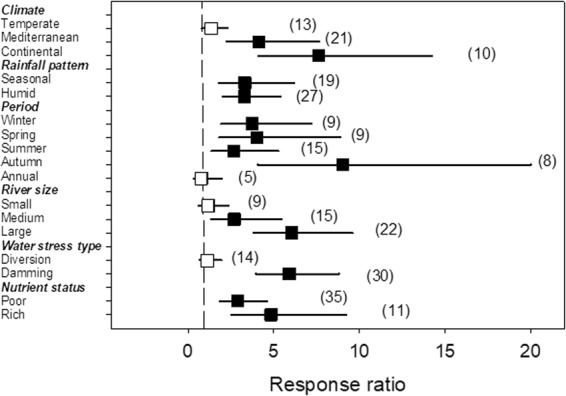
Effects of water stress on algal biomass as a function of climate, rainfall pattern, period of the year, river size, type of water stress and nutrient status, given by the response ratio (R = Impacted/Control; ±95% Confidence Limit, CL). The dashed line (mean effect size = 1) indicates no effect. Mean effect size >1 indicates an increase, while mean effect size <1 indicates a decrease due to water stress. The effect of water stress is significant when the 95% CL does not overlap 1 (black symbols). Levels within a given moderator (in bold) do not significantly differ when the 95% CL overlap. Values in the parentheses indicate sample size.

Algal biomass (chlorophyll-*a*) showed a large response to HDWS, with an average 3.30-fold increase (Fig. 1). Although the response of benthic algal biomass was highly positive (Fig. 3), its magnitude was modulated by the climate (p < 0.001; stronger for continental than temperate climate), period of the year (p = 0.009; stronger for autumn than annual periods), river size (p = 0.002; stronger for larger than smaller systems) and the type of water stress (p < 0.001; stronger in rivers regulated by dams than in those with flow diversion) (Table 2, Fig. 3).

**Table 2:** Datasets and moderators tested in the analyses with number of levels within moderators, total sample size, Rosenberg fail safe number (a dataset is robust to publication bias if Nfs >5 × n + 10, n = number of effect sizes) and Q_M_ statistics (significant differences among levels within moderators exist if p < 0.050).

Dataset	Moderator	No. levels	Total n	Rosenberg Nfs	Q_M_	df	p
**General Responses**							
All	Variable type	6	261	1121326	72.404	5	<0.001
Chemistry without Personal care products, Pesticides, Industrial compounds	Variable type 2	2	57	8234	22.146	1	<0.001
Nutrients excluding *Tot-N*	Variable	5	49	6918	3.093	4	0.542
Invertebrates	Variable	4	52	426676	4.995	3	0.172
Function	Variable type 2	2	55	38595	54.378	1	<0.001
Metabolism	Variable	2	14	1490	0.222	1	0.637
**Algae**							
Algae	Climate without *Tropical*	3	44	10724	13.051	2	0.001
Algae	Rainfall regime	2	46	11266	<0.001	1	0.991
Algae	Period of the year	5	46	11266	13.603	4	0.009
Algae	River size	3	46	11266	12.806	2	0.002
Algae	Water stress without *Channelization*	2	44	10708	22.159	1	<0.001
Algae	Nutrient status	2	46	11266	1.265	1	0.261
**Invertebrate richness**							
Invertebrate richness	Climate without *Continental*	2	23	202916	0.011	1	0.915
Invertebrate richness	Rainfall regime	3	24	208767	5.248	2	0.073
Invertebrate richness	Period of the year	3	21	11858	0.606	2	0.739
Invertebrate richness	River size without *Large rivers*	2	13	100636	2.317	1	0.128
Invertebrate richness	Water stress	4	23	202916	15.638	3	0.001
**Breakdown**							
Breakdown	Climate	3	41	30916	14.56	2	<0.001
Breakdown	Rainfall regime	2	41	30916	6.914	1	0.009
Breakdown	Period of the year	4	41	30916	7.853	3	0.049
Breakdown	Stream order	2	41	30916	29.082	1	<0.001
Breakdown	Water stress without *Natural*	2	40	30054	1.643	1	0.200
Breakdown	Nutrient status	2	41	30916	4.226	1	0.040

### Invertebrates

**Figure 4:**
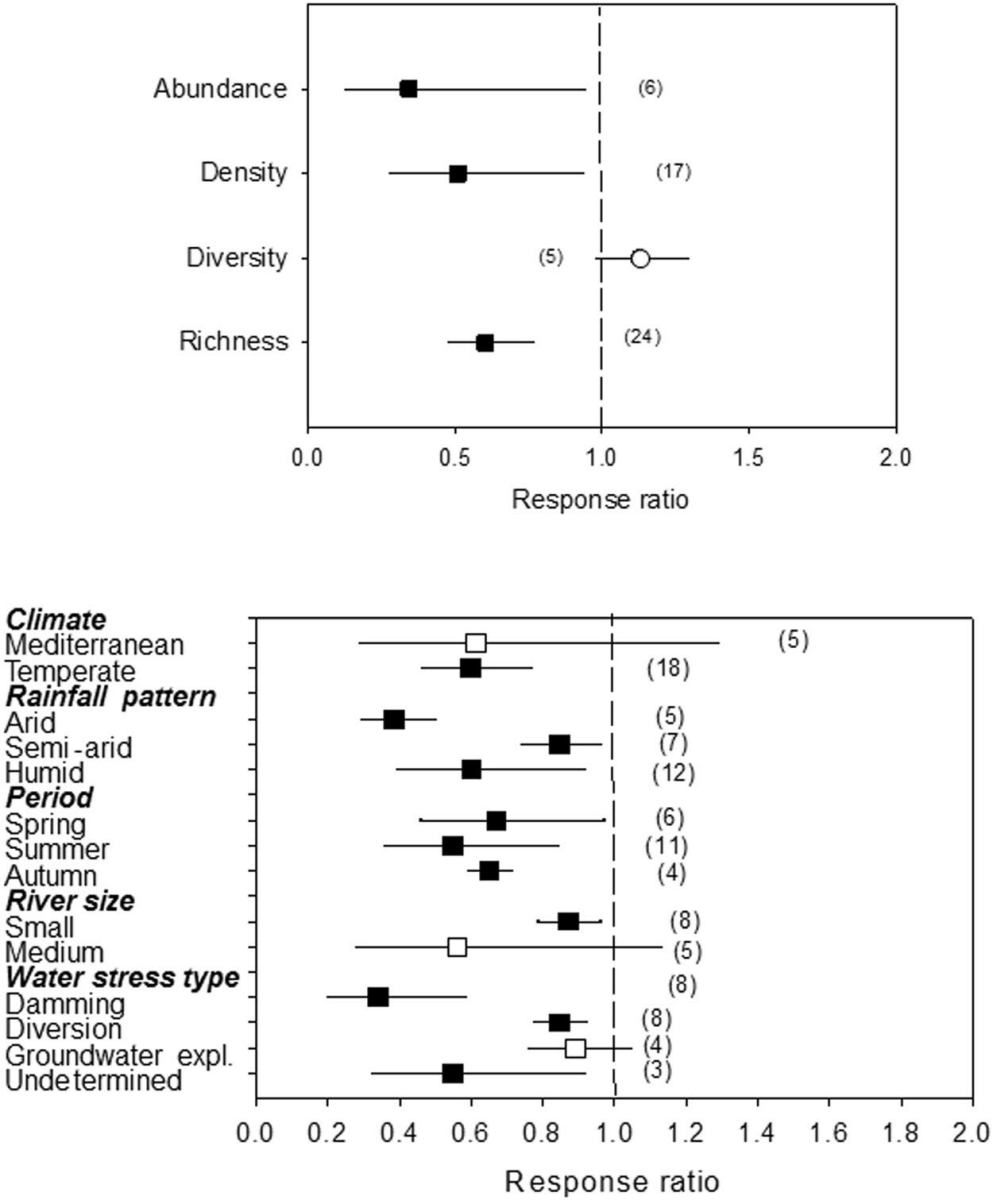
(Top) Effects of water stress on invertebrate abundance, density, diversity and richness. (Bottom) Effects of water stress on macroinvertebrate richness as a function of climate, rainfall pattern, period of the year, river size and the type of water stress, given by the response ratio (R = Impacted/Control; ±95% Confidence Limit, CL). The dashed line (mean effect size = 1) indicates no effect. Mean effect size >1 indicates an increase, while mean effect size <1 indicates a decrease due to water stress. The effect of water stress is significant when the 95% CL does not overlap 1 (black symbols). Levels within a given moderator (in bold) do not significantly differ when the 95% CL overlap. Values in the parentheses indicate sample size.

For invertebrates, abundance, density and richness were significantly reduced by HDWS (R = 0.34, 0.51 and 0.60, respectively; 95% CL: 0.13–0.95, 0.28–0.94 and 0.46–0.77, respectively), while diversity was not significantly affected (R = 1.28; 95% CL: 0.98–1.30; Fig. 4a). The response of invertebrate richness was negative overall (Fig. 4b), with its magnitude being higher in arid systems and depending on the type of water stress (p = 0.001; stronger in rivers regulated by dams than in those with flow diversion or groundwater exploitation) (Table 2).

### Ecosystem function

**Figure 5:**
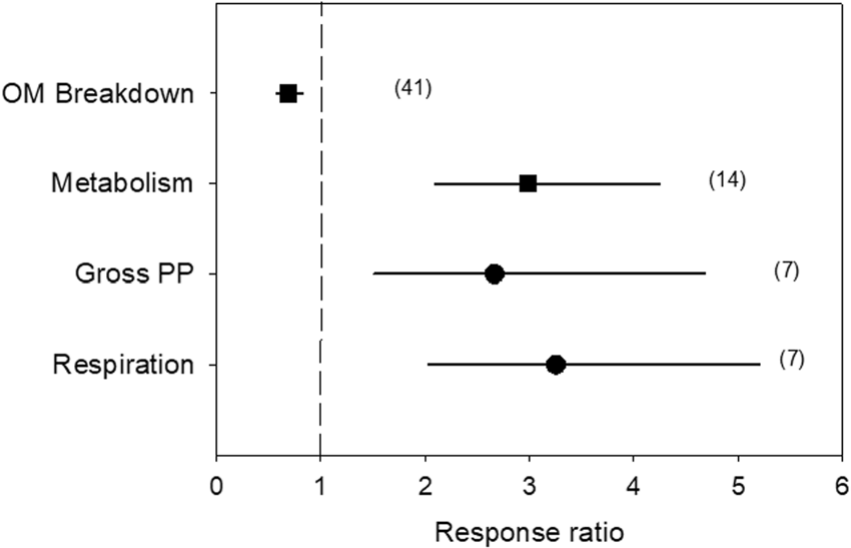
Effects of water stress on ecosystem functions (organic matter breakdown, gross primary production and respiration), given by the response ratio (R = Impacted/Control; ±95% Confidence Limit, CL). The dashed line (mean effect size = 1) indicates no effect. Mean effect size >1 indicates an increase, while mean effect size <1 indicates a decrease due to water stress. The effect of water stress is significant when the 95% CL does not overlap 1. Levels within a given moderator (same symbol) do not significantly differ when the 95% CL overlap. Values in the parentheses indicate sample size.

**Figure 6:**
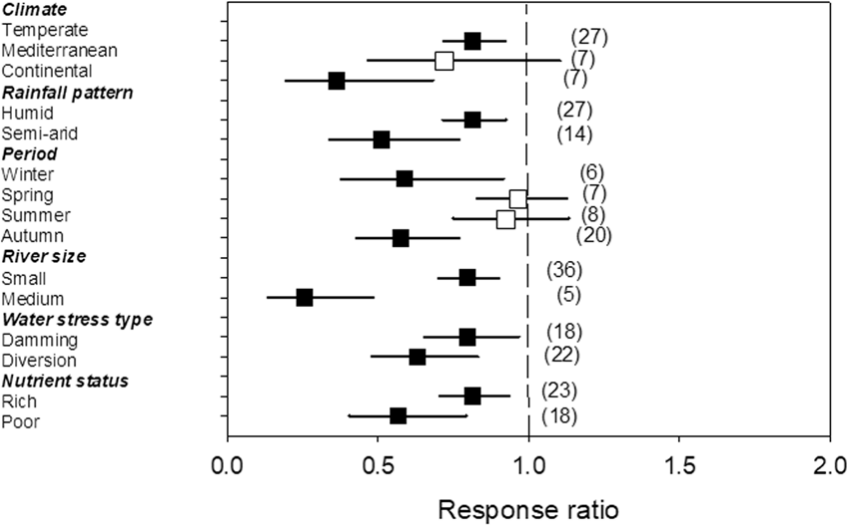
Effects of water stress on organic matter breakdown, as modulated by climate, rainfall pattern, period of the year, river size, type of water stress and nutrient status, given by the response ratio (R = Impacted/Control; ±95% Confidence Limit, CL). The dashed line (mean effect size = 1) indicates no effect. Mean effect size >1 indicates an increase, while mean effect size <1 indicates a decrease due to water stress. The effect of water stress is significant when the 95% CL does not overlap 1 (black symbols). Levels within a given moderator (in bold) do not significantly differ when the 95% CL overlap. Values in the parentheses indicate sample size.

Although ecosystem function as a whole did not show a general response to water stress (Fig. 1), stream metabolism was significantly enhanced by water stress (R = 2.99; 95% CL: 2.10–4.25), while organic matter breakdown was significantly reduced (R = 0.69; 95% CL: 0.58–0.82) (Fig. 5). When considering only stream metabolism, both the gross primary production and respiration were significantly, and similarly, increased by water stress (R = 2.67 and 3.25, respectively; 95% CL: 1.52–4.68 and 2.03–5.21, respectively) (Fig. 5). Although the response of organic matter breakdown to water stress was generally negative (Fig. 6), its magnitude depended on climate (p < 0.001; stronger for continental than temperate climate), rainfall regime (p = 0.009; stronger for seasonal than humid weather), period of the year (p = 0.049; stronger for autumn than for spring and summer), river size (p < 0.001; stronger for medium than for low order) and nutrient status (p = 0.040; stronger for nutrient-poor than nutrient-rich streams) (Table 2).

### Sensitivity analysis

When considering a mean effect size per study-variable combination, the significance and direction of the effects did not change substantially (Table S3) compared to those using the entire matrix (Figs 1–5). Only the effect on PO_4_ concentration (which was previously significant; R = 0.72; 95% CL: 0.54–0.98) became non-significant (R = 0.99; 95% CL: 0.65–1.51) probably due to the smaller sample size (n = 5). For invertebrates, the reduction in sample size (n = 3 and 7, respectively) caused the effect on abundance and density to become non-significant (R = 0.26 and 0.62, respectively; 95% CL: 0.03–2.12 and 0.36–1.08, respectively).

## Discussion

Our meta-analysis showed that HDWS has a strong impact on river ecosystems, affecting water chemistry, algal biomass, the abundance, density and richness of invertebrates, as well as ecosystem functioning. These effects occurred in sites affected by a general decrease in the average water flow and a lower variability in water flow regimes; this decrease, calculated for those studies with available hydrological data, was in average two thirds of the water flow circulating in the control sites (paired t-test, p = 0.06). Beyond that, the direction and extent of the effects, as well as the relevance of the moderator variables, differed among the response variables. The most relevant moderators were climate, rainfall regime, river size and the type of HDWS.

Water quality was only partly affected by water stress. The effects on nutrient and contaminant concentrations were constrained to only a few elements, and showed diverging responses. Our meta-analysis detected a decrease in phosphate concentration and an increase in the concentration of pharmaceutical products. Even for these two variables, the effect size and variability of the response ranged from moderate (27%) to extremely large (114-3,530%). These different patterns of response suggest contrasting response mechanisms. Reduced dilution would favour an increased concentration of contaminants^39^, whereas increased hydraulic retention time and elevated photolysis would promote contaminant degradation, but not necessarily their total elimination. Although this could not be tested in our meta-analysis, HDWS conditions could favour both higher concentrations as well as intense transformation of pollutants^40,41^. Moreover, higher biological activity in HDWS (^32^; see below) might influence the biogeochemical response of non-conservative solutes (e.g. phosphate) and contribute reducing their concentrations.

HDWS apparently homogenised the community structure of biological communities; however, the paucity of data for some groups of organisms (e.g., bacteria and fish) and variables (community composition and selected key species) limited our statistical power and prevented generalisation. Fish data, as an example, were available from only five different studies comparing the methods tested. Such high variability could have contributed to our failure in detecting significant effects of water stress on fish density. Nevertheless, several studies indicate that water stress affects fish assemblages; abstraction has been reported to influence assemblage composition in Mediterranean rivers affected by HDWS^42^, whereas salmonids have been shown to be sensitive to reduced levels of dissolved oxygen and higher water temperatures associated with HDWS^43^. Other studies suggest that water stress may modify fish behaviour and feeding habits^44^, but these effects should be confirmed with further data.

Water stress produced one of the clearest effects on benthic algal biomass. Algal biomass responded positively to water stress, mostly as a result of the steady hydrology associated to damming or water abstraction, which promotes biomass accumulation and decreases drift. Although we could not test for assemblage diversity or for specific effects on taxa, several studies suggest that water stress may affect both^45,46^. Our meta-analysis showed that algal biomass increased 1.5- to 10-fold in HDWS, the highest responses occurring in nutrient-rich sites, areas regulated by dams and larger river systems, mostly during spring and autumn. The larger effects on algal biomass could therefore occur in well-lit and nutrient-rich rivers affected by HDWS^47^. The accumulation of primary producers might in turn affect nutrient uptake and concentration^48^, causing the decrease in concentration of inorganic phosphorus and the non-significant effects on nitrogen compounds that we detected in the meta-analysis. The unexpected lower nutrient concentrations can be accounted for algal accumulation that actively depletes available nutrients, mostly during the most favorable periods for algal growth.

The meta-analysis also showed effects on the abundance, density and richness of invertebrate assemblages, which were all significantly reduced under water stress. Invertebrates are highly sensitive to the stable hydrological conditions that water stress promotes. Richness is greatly reduced both in regulated rivers^14,49,50^ and in those affected by water abstraction^16^. Our meta-analysis showed that this decrease in invertebrate richness occurred under nearly all types of water stress, especially in dry climate river systems and under dams. A surprising lack of effect in Mediterranean rivers could have been due to both the low number of data (n = 5) and the flow inversion during dry periods^51^. Higher water demand for irrigation that occurs in the regulated rivers of the region might unexpectedly enhance richness of higher invertebrates. Flow regulation by dams had the highest impact on invertebrates, especially filter feeders, grazers and shredders, while predators were unaffected^52^. This selective effect on trophic strategies probably correspond to the effects on Ephemeroptera, Plecoptera and Trichoptera taxa (EPT), which are the most sensitive to changing physical conditions^52^. Dam outflow patterns lead to unfavourable conditions for rheophile species^53,54^, since these have more specific requirements for respiration and feeding. In many situations, rheophiles are replaced by lentic species^30^ or by taxa more tolerant to the new conditions^55^. Other changes associated with water stress may explain the lower abundance and density of invertebrates in HDWS, including a higher frequency of pupation and a faster emergence and drift^56,57^.

Water stress elicited a 3-fold increase in downstream river metabolism. Metabolism estimates energy fluxes as gross primary production (GPP) and respiration (R) in a river. We could assemble only 7 data of metabolism, including data on open channels and chamber measurements, which probably caused large variability in the data. Despite this, both GPP and R were strongly enhanced by water stress (2.67-fold and 3.25-fold, respectively), possibly as a response to the accumulation of organic matter (both autochthonous and allochthonous) under steady flows^32^. However, the wetted-channel contraction following water loss in HDWS would not only produce effects on a per-surface-unit basis, but also on a per-unit-of-channel-length basis^13^, leading to an overall decrease of the production and organic matter processing within the channel. Further research is required to determine the implications of changes in metabolism elicited by HDWS for the riverine food web and nutrient dynamics at the ecosystem scale.

Finally, our analysis revealed that water stress reduced organic matter breakdown by an average of 31%. The reduction was greater in areas with a continental climate, during autumn and winter, and in medium-sized rivers, mostly in nutrient-poor conditions. This suggests that the effects of water stress on organic matter breakdown might be important in shaded river ecosystems during periods of maximum inputs of organic matter. Organic matter breakdown results from a combination of physical fragmentation and the activities of microorganisms (bacteria and fungi) and detritivores^58^. Decreased organic matter breakdown under water stress can result from lower physical abrasion^59^ and reduced fungal biomass^60^, which may slow down the breakdown of large macromolecules^61^. The effects of HDWS on the abundance and biomass of shredders might also contribute to a slower degradation of organic matter^62^. The observed non-significant effect of water stress on organic matter breakdown in spring and summer could be related to the natural reduction in shredder abundance during these periods, when most develop into adults^63^. Furthermore, less diverse and less abundant shredder communities could render organic matter breakdown less sensitive to HDWS in Mediterranean rivers. This link between the smaller presence of shredders and poor organic matter decomposition under HDWS is exemplified in rivers in New Zealand^64^, where effects of reduced discharge on organic matter decomposition is low and occur in the absence of specialized shredders.

The meta-analysis showed that HDWS induced a wide range of effects on the structure and function of river ecosystems, which did not match those occurring naturally in temporary rivers^65^. The effect diverged in intensity according to the type of water stress, with dams causing the strongest effects, followed by water diversion and channelization, being groundwater extraction the weakest. Our results have implications beyond the local scale because the extent of regulation and water abstraction in some river systems could have a general effect on river networks. The Sacramento River shows a frequency of 1.4 diversion points per linear kilometre^57^, while diversion canals as well as small and large dams affect most temperate, semi-arid and arid river networks^16,30^. Water stress therefore occurs at river segment and ultimately at watershed scales, and their effects may constitute a phenomenon in many world regions.

Our study emphasises some of the effects of HDWS on rivers, but does not account for others due to low sample sizes. There is a scarcity in the number of studies directly addressing the effects of HDWS. Consequences of HDWS on food webs are still unknown (but see^33,66^), while implications for keystone species in the ecosystem can only be speculated. Data on microbial organisms (essential contributors to the energy flux of river ecosystems) are mostly restricted to autochthonous species, with little information on bacteria or fungi^67^. Although a large amount of data were assembled that indicated a clear effect of water stress on river structure and function, expanding our knowledge to fill these gaps is an essential step in forecasting the impact of water stress on river ecosystems.

## Electronic supplementary material

Table S1

Dataset 1

Table S3
