# Supplementary material for: Effects of human-driven water stress on river ecosystems: a meta-analysis
Source: Sci Rep. 2018 Jul 30;8:11462. doi: 10.1038/s41598-018-29807-7 (PMC6065398; doi:10.1038/s41598-018-29807-7)
Supplement: Supplementary file 1 — Table S1 [file 41598_2018_29807_MOESM1_ESM.docx]

**Table S1. List of primary studies included in the review.**

**Effects of human-driven water stress on river ecosystems: a meta-analysis**

**Sergi Sabater^1,2^*****, Francesco Bregoli^1,3^, Vicenç Acuña^1^, Damià Barceló^1,4^, Arturo Elosegi^5^, Antoni Ginebreda^4^, Rafael Marcé^1^, Isabel Muñoz^6^, Laia Sabater-Liesa^4^, Verónica Ferreira^7^**

1- Catalan Institute for Water Research (ICRA). Carrer Emili Grahit 101, 17003 Girona, Spain

2- Institute of Aquatic Ecology, University of Girona, Campus Montilivi, 17071 Girona, Spain

3- Water Science and Engineering Department, IHE Delft Institute for Water Education, Westvest 7, 2611 AX Delft, The Netherlands

4-Department of Environmental Chemistry, Institute of Environmental Assessment and Water Research (IDAEA-CSIC), Carrer Jordi Girona 18-26, 08034 Barcelona, Spain

5-Laboratory of Stream Ecology, Department of Plant Biology and Ecology, University of the Basque Country, 48080 Bilbao (Spain)

6- Department of Evolutionary Biology, Ecology and Environmental Sciences, Universitat de Barcelona, Avgda. Diagonal 643, 08028, Barcelona, Spain

7- MARE – Marine and Environmental Sciences Centre, Department of Life Sciences, University of Coimbra, 3004–517, Coimbra, Portugal

*Correspondence author: sergi.sabater@udg.edu; Tel. +34 972 183 380; Fax. +34 972 183 248.

1 Abril, M. *et al.* Effects of water flow regulation on ecosystem functioning in a Mediterranean river network assessed by wood decomposition. *Science of the Total Environment* **517**, 57-65 (2015).

2 Abril, M., Muñoz, I. & Menéndez, M. Heterogeneity in leaf litter decomposition in a temporary Mediterranean stream during flow fragmentation. *Science of the Total Environment* **553**, 330-339 (2016).

3 Aristi, I. *et al.* Flow regulation by dams affects ecosystem metabolism in Mediterranean rivers. *Freshwater biology* **59**, 1816-1829 (2014).

4 Arroita, M. *et al.* Impact of water abstraction on storage and breakdown of coarse organic matter in mountain streams. *Science of the Total Environment* **503**, 233-240 (2015).

5 Arroita, M. *et al.* Water abstraction impacts stream ecosystem functioning via wetted-channel contraction. *Freshwater Biology* **62**, 243-257, doi:10.1111/fwb.12864 (2017).

6 Badr, E.-S. A. Spatio-temporal variability of dissolved organic nitrogen (DON), carbon (DOC), and nutrients in the Nile River, Egypt. *Environ Monit Assess ()* **188**, 580 (2016).

7 Benejam, L., Angermeier, P. L., Munne, A. & Garcia-Berthou, E. Assessing effects of water abstraction on fish assemblages in Mediterranean streams. *Freshwater Biology* **55**, 628-642, doi:10.1111/j.1365-2427.2009.02299.x (2010).

8 Bogan, M. T., Boersma, K. S. & Lytle, D. A. Flow intermittency alters longitudinal patterns of invertebrate diversity and assemblage composition in an arid‐land stream network. *Freshwater Biology* **58**, 1016-1028 (2013).

9 Chessman, B. C., Royal, M. J. & Muschal, M. The challenge of monitoring impacts of water abstraction on macroinvertebrate assemblages in unregulated streams. *River Res. Appl.* **27**, 76-86, doi:10.1002/rra.1340 (2011).

10 Collado, N. *et al.* Pharmaceuticals occurrence in a WWTP with significant industrial contribution and its input into the river system. *Environmental Pollution* **185**, 202-212 (2014).

11 De Merona, B. & Albert, P. Ecological monitoring of fish assemblages downstream of a hydroelectric dam in French Guiana (South America). *Regulated Rivers-Research & Management* **15**, 339-351, doi:10.1002/(sici)1099-1646(199907/08)15:4<339::aid-rrr546>3.0.co;2-0 (1999).

12 Death, R. G., Dewson, Z. S. & James, A. B. Is structure or function a better measure of the effects of water abstraction on ecosystem integrity? *Freshwater Biology* **54**, 2037-2050 (2009).

13 Dewson, Z. S., James, A. B. W. & Death, R. G. Stream ecosystem functioning under reduced flow conditions. *Ecological Applications* **17**, 1797-1808, doi:10.1890/06-1901.1 (2007).

14 Fairbairn, D. J. *et al.* Sources and transport of contaminants of emerging concern: A two-year study of occurrence and spatiotemporal variation in a mixed land use watershed. *Science of the Total Environment* **551**, 605-613 (2016).

15 Fantin-Cruz, I., Pedrollo, O., Girard, P., Zeilhofer, P. & Hamilton, S. K. Changes in river water quality caused by a diversion hydropower dam bordering the Pantanal floodplain. *Hydrobiologia* **768**, 223-238 (2016).

16 Fayolle, S., Cazaubon, A., Comte, K. & Franquet, E. The Intermediate Disturbance Hypothesis: application of this concept to the response of epilithon in a regulated Mediterranean river (Lower-Durance, southeastern France). *Archiv Fur Hydrobiologie* **143**, 57-77 (1998).

17 Fenoglio, S., Bo, T., Cucco, M. & Malacarne, G. Response of benthic invertebrate assemblages to varying drought conditions in the Po river (NW Italy). *Italian Journal of Zoology* **74**, 191-201 (2007).

18 Finn, M. A., Boulton, A. J. & Chessman, B. C. Ecological responses to artificial drought in two Australian rivers with differing water extraction. *Fundamental and Applied Limnology* **175**, 231-248, doi:10.1127/1863-9135/2009/0175-0231 (2009).

19 Gehrke, P., Brown, P., Schiller, C., Moffatt, D. & Bruce, A. River regulation and fish communities in the Murray‐Darling river system, Australia. *River Res. Appl.* **11**, 363-375 (1995).

20 Gehrke, P. C., Gilligan, D. M. & Barwick, M. Changes in fish communities of the Shoalhaven River 20 years after construction of Tallowa Dam, Australia. *River Res. Appl.* **18**, 265-286, doi:10.1002/rra.669 (2002).

21 Hax, C. L. & Golladay, S. W. Flow disturbance of macroinvertebrates inhabiting sediments and woody debris in a prairie stream. *American Midland Naturalist. Apr* **139**, 210-223 (1998).

22 Hill, B., Gardner, T. & Ekisola, O. Breakdown of gallery forest leaf litter in intermittent and perennial prairie streams. *The Southwestern Naturalist*, 323-331 (1988).

23 Jellyman, P. G. & Harding, J. S. The role of dams in altering freshwater fish communities in New Zealand, New Zealand. *Journal of Marine and Freshwater Research* **46**, 475-489 (2012).

24 Lessard, J. *et al.* Dam design can impede adaptive management of environmental flows: a case study from the Opuha Dam, New Zealand. *Environmental management* **51**, 459-473 (2013).

25 Mackie, J. K., Chester, E. T., Matthews, T. G. & Robson, B. J. Macroinvertebrate response to environmental flows in headwater streams in western Victoria, Australia. *Ecological engineering* **53**, 100-105 (2013).

26 Mariluan, G. D., Villanueva, V. D. & Albariño, R. J. Leaf litter breakdown and benthic invertebrate colonization affected by seasonal drought in headwater lotic systems of Andean Patagonia. *Hydrobiologia* **760**, 171-187 (2015).

27 Martínez, A. *et al.* Stream regulation by small dams affects benthic macroinvertebrate communities: from structural changes to functional implications. *Hydrobiologia* **711**, 31-42 (2013).

28 Matthaei, C. D., Piggott, J. J. & Townsend, C. R. Multiple stressors in agricultural streams: interactions among sediment addition, nutrient enrichment and water abstraction. *Journal of Applied Ecology* **47**, 639-649, doi:10.1111/j.1365-2664.2010.01809.x (2010).

29 McKay, S. F. & King, A. J. Potential ecological effects of water extraction in small, unregulated streams. *River Res. Appl.* **22**, 1023-1037, doi:10.1002/rra.958 (2006).

30 Mendoza–Lera, C. *et al.* Headwater reservoirs weaken terrestrial‐aquatic linkage by slowing leaf‐litter processing in downstream regulated reaches. *River Res. Appl.* **28**, 13-22 (2012).

31 Menéndez, M., Descals, E., Riera, T. & Moya, O. Effect of small reservoirs on leaf litter decomposition in Mediterranean headwater streams. *Hydrobiologia* **691**, 135-146 (2012).

32 Merciai , R., Molons-Sierra, C., Sabater, S. & García-Berthou, E. Water abstraction affects abundance, size-structure and growth of two threatened cyprinid fishes. *PlosOne* **12**, e017593.017592 (2017).

33 Miserendino, M. L. Effects of flow regulation, basin characteristics and land-use on macroinvertebrate communities in a large arid Patagonian river. *Biodiversity and Conservation* **18**, 1921-1943, doi:10.1007/s10531-008-9565-3 (2009).

34 Munn, M. D. & Brusven, M. A. The influence of Dworshak Dam on epilithic community metabolism in the Clearwater River, USA. *Hydrobiologia* **513**, 121-127, doi:10.1023/B:hydr.0000018177.78841.08 (2004).

35 Pinna, M. *et al.* Influence of aperiodic summer droughts on leaf litter breakdown and macroinvertebrate assemblages: testing the drying memory in a Central Apennines River (Aterno River, Italy). *Hydrobiologia* **782**, 111-126 (2016).

36 Ponsatí, L. *et al.* Biofilm Responses to Flow Regulation by Dams in Mediterranean Rivers. *River Res. Appl.* **31**, 1003-1016, doi:10.1002/rra.2807 (2015).

37 Proia, L. *et al.* Drought episode modulates the response of river biofilms to triclosan. *Aquatic Toxicology* **127**, 36-45, doi:10.1016/j.aquatox.2012.01.006 (2013).

38 Roberts, J. *et al.* Pharmaceuticals and personal care products (PPCPs) in Australia's largest inland sewage treatment plant, and its contribution to a major Australian river during high and low flow. *Science of the Total Environment* **541**, 1625-1637 (2016).

39 Santucci, V. J., Gephard, S. R. & Pescitelli, S. M. Effects of Multiple Low‐Head Dams on Fish, Macroinvertebrates, Habitat, and Water Quality in the Fox River, Illinois. *North American Journal of Fisheries Management* **25**, 975-992 (2005).

40 Skoulikidis, N. T. *et al.* Assessing water stress in Mediterranean lotic systems: insights from an artificially intermittent river in Greece. *Aquatic Sciences* **73**, 581-597, doi:10.1007/s00027-011-0228-1 (2011).

41 Solagaistua, L., Arroita, M., Aristi, I., Larrañaga, A. & Elosegi, A. Changes in discharge affect more surface than subsurface breakdown of organic matter in a mountain stream. *Marine and Freshwater Research* **67**, 1826-1834 (2016).

42 Verdonschot, R., Oosten‐Siedlecka, A. M., Braak, C. J. & Verdonschot, P. F. Macroinvertebrate survival during cessation of flow and streambed drying in a lowland stream. *Freshwater Biology* **60**, 282-296 (2015).

43 White, H. L., Nichols, S. J., Robinson, W. A. & Norris, R. H. More for less: a study of environmental flows during drought in two Australian rivers. *Freshwater Biology* **57**, 858-873 (2012).

44 Wooster, D., Miller, S. W. & DeBano, S. J. Impact of season-long water abstraction on invertebrate drift composition and concentration. *Hydrobiologia* **772**, 15-30 (2016).
