# Supplementary material for: Effects of human-driven water stress on river ecosystems: a meta-analysis
Source: Sci Rep. 2018 Jul 30;8:11462. doi: 10.1038/s41598-018-29807-7 (PMC6065398; doi:10.1038/s41598-018-29807-7)
Supplement: Supplementary file 3 — Table S3 [file 41598_2018_29807_MOESM3_ESM.docx]

**Table S3. Summary table for subgroup analyses considering a mean effect size per study-variable combination.**

**Effects of human-driven water stress on river ecosystems: a meta-analysis**

**Sergi Sabater^1,2^*****, Francesco Bregoli^1,3^, Vicenç Acuña^1^, Damià Barceló^1,4^, Arturo Elosegi^5^, Antoni Ginebreda^4^, Rafael Marcé^1^, Isabel Muñoz^6^, Laia Sabater-Liesa^4^, Verónica Ferreira^7^**

1- Catalan Institute for Water Research (ICRA). Carrer Emili Grahit 101, 17003 Girona, Spain

2- Institute of Aquatic Ecology, University of Girona, Campus Montilivi, 17071 Girona, Spain

3- Water Science and Engineering Department, IHE Delft Institute for Water Education, Westvest 7, 2611 AX Delft, The Netherlands

4-Department of Environmental Chemistry, Institute of Environmental Assessment and Water Research (IDAEA-CSIC), Carrer Jordi Girona 18-26, 08034 Barcelona, Spain

5-Laboratory of Stream Ecology, Department of Plant Biology and Ecology, University of the Basque Country, 48080 Bilbao (Spain)

6- Department of Evolutionary Biology, Ecology and Environmental Sciences, Universitat de Barcelona, Avgda. Diagonal 643, 08028, Barcelona, Spain

7- MARE – Marine and Environmental Sciences Centre, Department of Life Sciences, University of Coimbra, 3004–517, Coimbra, Portugal

*Correspondence author: sergi.sabater@udg.edu; Tel. +34 972 183 380; Fax. +34 972 183 248.

Table S3. Summary table for subgroup analyses considering a mean effect size per study-variable combination. Rosenberg's fail safe number for subgroups, sample size (n), mean effect size R, 95% CL and p value for levels within subgroups, test for heterogeneity between levels of moderators (Q), degrees of freedom (df) and p value are shown.

| Dataset | Levels | R | Lower 95%CL | Upper 95%CL | n | I2 | p |
| --- | --- | --- | --- | --- | --- | --- | --- |
| All | Overall | 0.981 | 0.804 | 1.197 | 85 | 98.85 | <0.001 |
|  | Chemistry | 1.181 | 0.910 | 1.531 | 27 | 97.98 | <0.001 |
|  | Bacteria | 1.003 | 0.649 | 1.550 | 3 | 80.29 | 0.022 |
|  | Algae | 2.022 | 1.065 | 3.838 | 12 | 94.23 | <0.001 |
|  | Invertebrates | 0.561 | 0.377 | 0.835 | 21 | 98.98 | <0.001 |
|  | Fish | 0.646 | 0.368 | 1.134 | 8 | 97.59 | <0.001 |
|  | Function | 1.116 | 0.741 | 1.684 | 14 | 94.15 | <0.001 |
|  | QM = 20.992, df = 5, p-val < 0.001 | |  |  |  |  |  |
|  |  |  |  |  |  |  |  |
| Nutrients without DIN, SRP, Tot-N | NH4 | 1.501 | 0.899 | 2.509 | 5 | 93.73 | <0.001 |
| Nfs = 317 | NO3 | 0.856 | 0.466 | 1.575 | 6 | 98.42 | <0.001 |
|  | PO4 | 0.993 | 0.653 | 1.510 | 5 | 92.68 | <0.001 |
|  | Tot-P | 1.019 | 0.560 | 1.855 | 3 | 93.63 | <0.001 |
|  | QM = 2.334, df = 3, p-val = 0.506 | |  |  |  |  |  |
|  |  |  |  |  |  |  |  |
| Invertebrates | Abundance | 0.261 | 0.032 | 2.106 | 3 | 98.25 | <0.001 |
| Nfs = 1769 | Density | 0.624 | 0.358 | 1.084 | 7 | 94.44 | <0.001 |
|  | Richness | 0.606 | 0.390 | 0.941 | 9 | 98.77 | <0.001 |
|  | QM = 2.309, df = 2, p-val = 0.315 | |  |  |  |  |  |
|  |  |  |  |  |  |  |  |
| Function | Metabolism | 2.440 | 1.545 | 3.857 | 6 | 66.62 | 0.009 |
| Nfs = 29 | Breakdown | 0.693 | 0.546 | 0.881 | 8 | 78.81 | 0.001 |
|  | QM = 27.603, df = 1, p-val < 0.001 | |  |  |  |  |  |
|  |  |  |  |  |  |  |  |
| Metabolism | GPP | 2.226 | 1.074 | 4.614 | 3 | 71.51 | 0.028 |
| Nfs = 83 | ER | 2.664 | 1.334 | 5.317 | 3 | 71.75 | 0.024 |
|  | QM = 0.123, df = 1, p-val = 0.726 | |  |  |  |  |  |
|  |  |  |  |  |  |  |  |
|  |  |  |  |  |  |  |  |
